# Mechanism and toxicity evaluation of catalytic ozonation over Cu/Ce–Al_2_O_3_ system aiming at degradation of humic acid in real wastewater

**DOI:** 10.1038/s41598-021-83804-x

**Published:** 2021-04-22

**Authors:** Xi Tang, Yifei Zhang, Weiqi Li, Jinju Geng, Hongqiang Ren, Ke Xu

**Affiliations:** State Key Laboratory of Pollution Control and Resource Reuse, School of the Environment, Nanjing University, 163 Xianlin Avenue, Qixia District, Nanjing, 210023 Jiangsu People’s Republic of China

**Keywords:** Environmental sciences, Health care, Chemistry, Engineering

## Abstract

Humic acid (HA) is the main component of organic matter in effluent from wastewater treatment. The effective removal of HA is significant. In this study, a novel catalyst was prepared using a transition metal oxide as the active component and Al_2_O_3_ as a granular carrier. The mechanism of catalytic ozonation of HA under neutral pH conditions and its efficiency were investigated. Under the chosen conditions (an ozone concentration of 2.2 mg/L, 50 mg/L HA solution, catalyst dosage of 5 g/L and initial pH of 6.49), the Cu/Ce–Al_2_O_3_ bimetallic catalyst led to 54.79% TOC removal rate after 30 min; the removal rate by ozone alone was only 20.49%. The characteristics of organic compounds determined by FT-IR and GC–MS showed that organic compounds were degraded significantly by the catalytic treatment. The addition of catalysts could effectively degrade toxic intermediates and reduce the acute toxicity produced by ozonation. Humic acid substances were largely removed and transformed into biodegradable intermediates. This study proposes a new and efficient ozonation catalyst for practical applications in advanced wastewater treatment.

## Introduction

Advanced treatment and reuse of wastewater are an important contribution to integrated water resource management. In general, effluent organic matter (EfOM) from biological wastewater treatment contains natural organic matter (NOM), soluble microbial products (SMPs) and traces of harmful chemicals^1^. Proteins, humic acids (HAs) and carbohydrates are the three main components of EfOM^2^. For example, 40–50% of the components of EfOM in the effluent from a typical biological wastewater treatment plant were found to be humic substances^3^. Other studies showed that aquatic humic substances (AHS) and hydrophilic acidic substances (HPI-A) were the main components of EfOM; the average content of AHS in dissolved organic carbon (DOC) exceeded 55%^4^.

HA is a subclass of AHS. HA has a complex structure and contains functional groups such as carboxylic, phenolic, carbonyl, hydroxyl, aldehyde acid, and methoxyl^5^. The existence of carboxylic and phenolic groups causes HA to carry a negative charge in water. The conformation and aggregation states of HA are influenced by solution conditions such as the concentration, pH and ionic strength^6,7^. Previous studies have reported that HA is difficult to remove using conventional treatment processes, including coagulation, granular activated carbon (GAC) adsorption, and membrane filtration, with TOC removal efficiencies of only approximately 10–50%^8^.

As one of the advanced oxidation processes (AOPs), heterogeneous catalytic ozonation has received extensive attention for its ability to remove refractory organic compounds^9,10^. Many catalysts have been reported, including metal oxides^11–13^, supported metal or metal oxides^14–17^. However, the nano/micrometer scale of powder-like catalysts makes it difficult to separate the catalyst from the reaction mixture for reuse. Furthermore, the mechanism of the catalytic ozonation process for pollutant degradation remains ambiguous. Previous studies of the mechanism suggested that catalytic ozonation in the presence of metal oxides can proceed through two pathways: (1) the promotion of hydroxyl radicals generated from aqueous ozone and (2) surface complex formation between carboxylic groups of the pollutants and surface metal sites of the catalysts^9,10^. Studies on the removal effect and mechanism of catalytic ozonation on HA are relatively rare. The surface properties of catalysts greatly influence the removal efficiency, including the isoelectric point, specific surface area and Lewis acid site. The aforementioned is caused by the adsorption of ozone and organic compounds on the catalyst surface, which is the requisite step for catalytic ozonation^18^. Meanwhile, the functional groups and complex structure of HA can affect its removal efficiency by surface reactions, which cause HA to become negatively charged. Recently, bimetallic catalysts have received increased attention for their improved catalytic activity compared to that of single metal oxide catalysts^14,17^. Copper oxides (CuO_x_) have been proven to be effective catalysts for catalytic ozonation by increasing the density of surface hydroxyl groups and isoelectric points^13,18^. Cerium oxides (CeOx) have also been regarded as promising active catalysts for catalytic ozonation^11,12^. Cerium stores and releases oxygen through oxygen vacancy formation, thus enhancing its catalytic effects for ozone decomposition. However, the combination of Cu and Ce species as catalysts has not previously been reported for catalytic ozonation of HA. A thorough exploration of the reaction mechanism of Cu–Ce oxide-catalyzed ozonation of HA is essential.

Many studies have shown good results under acidic and alkaline conditions, but the effluent of the secondary clarifier is usually neutral in practical applications, hence results are needed under neutral conditions^14,15^. Meanwhile, the activity of the catalyst should be confirmed in natural water after initial experiments confirming catalytic activity in model solutions^10^. To date, little information has been reported on the removal efficiency of HA with ozonation catalysts in real wastewater. The correlation between removal efficiency of HA and other indicators by catalytic ozonation in wastewater is unknown. Qualitative analysis and toxicity evaluation of intermediate products are necessary to test the potential of practical application of catalytic ozonation^19^. While many studies on catalytic ozonation of HA focus on the potential energy of disinfection by-products, evaluation of acute toxicity has received little attention as an important indicator of the potential of catalytic ozonation in wastewater reuse ^20^.

In this study, preparation conditions including the choice of granular carrier, active component, impregnation concentration and compound concentration were optimized. Multiple techniques, including N_2_ adsorption/desorption, XRD, zeta potential, SEM, ICP-OES and XPS, were employed to characterize the composite catalyst. The effect of pH and ozone dosage on the catalytic ozonation of HA was investigated. The catalytic mechanism was revealed by adding tert-butyl alcohol (TBA) and phosphate. Moreover, the catalytic stability and biochemical toxicity of the catalysts were evaluated. The characteristics of organic compounds before and after catalytic ozonation and sole ozonation were characterized by FT-IR and GC–MS. Four real wastewater samples from secondary sedimentation effluents from four WTTPs were used to examine the degradation efficiency of HA by catalytic ozonation in real wastewater.

## Experimental

### Chemicals and reagents

All chemicals were of analytical grade. Humic acid (HA) was purchased from Aladdin Reagent Co. (Shanghai, China). All other reagents were from Sinopharm Chemical Reagent Co. Ltd. (Shanghai, China). A humic acid (HA) stock solution was prepared by mixing 400 mg of HA with 1 L of ultrapure water in an ultrasonic cleaner at 40 kHz for 10 min. Then, a 50 mg/L HA solution was derived from the stock solution by dilution and filtering through a 0.45 μm membrane filter. The 50 mg/L HA solution had the following characteristics: DOC of 18.1 mg/L, UV_254_ of 1.611 cm^−1^, SUVA_254_ of 8.9 L/m mg, chroma of 378 and pH of 6.491. A 0.1 M hydrochloric acid (HCl) solution and 0.1 M sodium hydroxide (NaOH) solution were used for pH adjustment. The details about preparation and characterization methods of catalysts are shown in the supporting information.

Four real wastewater samples (WTTP1, WTTP2, WTTP3 and WTTP4), which were collected from secondary sedimentation effluents from four WTTPs, were used to test the effect of catalytic ozonation of HA in real wastewater. All three samples were filtered through a 0.45 mm cellulose nitrate membrane prior to use. The wastewater quality parameters are summarized in Table S1.

### Catalytic ozonation procedure

Ozone was produced by an ozonizer (WH-H-Y, Wohuan Ozone Mechanical and Electrical Equipment Company, China) with pure oxygen. The oxygen-ozone mixture was added to the solution through a microporous titanium plate at the bottom of a cylindrical plexiglass reactor at a flow rate of 400 mL/min. The working volume of the reactor was 1 L. The catalyst dosage was maintained at 5 g/L according to the preliminary experiment which could be seen in Fig S1, and the experiment was conducted at room temperature. The reaction time was 30 min. The ozone concentration in the solution was maintained at 2.90 mg/L. Specifically, the O_3_ produced during the first 4 min was discarded before the experimental operation to obtain a stable ozone flow. Samples were withdrawn at intervals, and sodium thiosulfate was used to quench the residual ozone in the sample. Then, the samples were filtered through a 0.45 μm Teflon filter for analysis. The catalytic stability of the catalyst was tested by washing the catalysts with deionized water and drying them at 105 °C, and then the same experimental procedure was followed.

### Analytical procedures

The concentration of ozone in the liquid phase was obtained by a potentiostatic controller for free chlorine, chlorine dioxide, and dissolved ozone (BC-CL7685, B&C Electronics, Italy). The TOC was analyzed by a combustion-type TOC analyzer (multi C/N 3100, Analytik Jena, Germany). The UV_254_ and chroma were quantified by an ultraviolet–visible spectrophotometer (UV2450, Shimadzu, Japan), COD, BOD were analyzed according to the Chinese State Environmental Protection Administration (SEPA) standard methods. The concentration of HA was measured by the improved Lowry method^21^. The concentration of leached Cu and Ce in the solution was determined by a flame atomic absorption spectrophotometer (MK & M6, Thermo). The water sample was freeze-dried using a lyophilizer (Lacbconco, USA) and the FT-IR spectra were analyzed with a Nexus 870 Fourier transform infrared spectrometer (Nicolet, USA). The compounds identified were determined by a 7890B gas chromatograph (Agilent, USA) interfaced to a mass selective detector (MSD). The method for determining biological toxicity of treated water samples was based on "Water quality—acute toxicity determination—luminescent bacteria method" (GB/T15441-1995). The details of the GC–MS analysis procedure and the determination of acute toxicity can be seen in supporting information.

### Statistical analysis

Statistical differences were evaluated using one-way analysis of variance (ANOVA) to determine whether the differences were significant or not. All analyses were performed using the SPSS statistical software package (SPSS Inc., U.S.A.).

## Results and discussion

### Characterization of the catalysts

Three copper catalysts were prepared using alumina (α-Al_2_O_3_), ceramic grain filters (CGF) and molecular sieves (MS) as carriers. SEM analysis was performed to observe the morphology of Al_2_O_3_, Cu-Al_2_O_3_ and Cu/Ce–Al_2_O_3_ (Fig. S2). It was important that the mix of Cu, Ce and Al_2_O_3_ did not change the structure of Al_2_O_3_ so as to maintain its duct and surface properties.

N_2_ adsorption–desorption was used to explore the structural properties of the samples. The zeta-potential was used to determine the surface isoelectric point and ICP-OES was used to measure the metal content in the metal-loaded catalyst. As shown in Table 1, the Al_2_O_3_ metal-loaded catalysts and Cu-MS exhibited a large specific surface area and large average pore diameter, which was beneficial to the surface interaction at the solid–liquid interface, while Cu-CGF had a small specific surface area and small average diameter. The pH_PZC_ of Cu/Ce–Al_2_O_3_ was the highest among these catalysts. Cu-MS had the highest metal loading rate of 5.41%.

**Table 1:** Physical properties of the catalysts.

Samples	BET surface area (m^2^/g)	Total pore volume (cm^3^/g)	Average pore diameter (nm)	pH_PZC_	Metal wt% (ICP)
Cu-GCF	0.5229	0.01	1.00	2.6	1.05
Cu-MS	441.5	0.12	1.09	2.2	5.41
Al_2_O_3_	180.03	0.45	9.78	9.5	/
Cu–Al_2_O_3_	129.5	0.39	11.91	9.9	2.15
Fe–Al_2_O_3_	131.9	0.39	11.86	9.7	3.67
Mn–Al_2_O_3_	141.8	0.40	11.48	9.8	1.27
Cu/Ce–Al_2_O_3_	149.4	0.34	9.14	10.2	2.00 (Cu) 0.63 (Ce)

**Figure 1:**
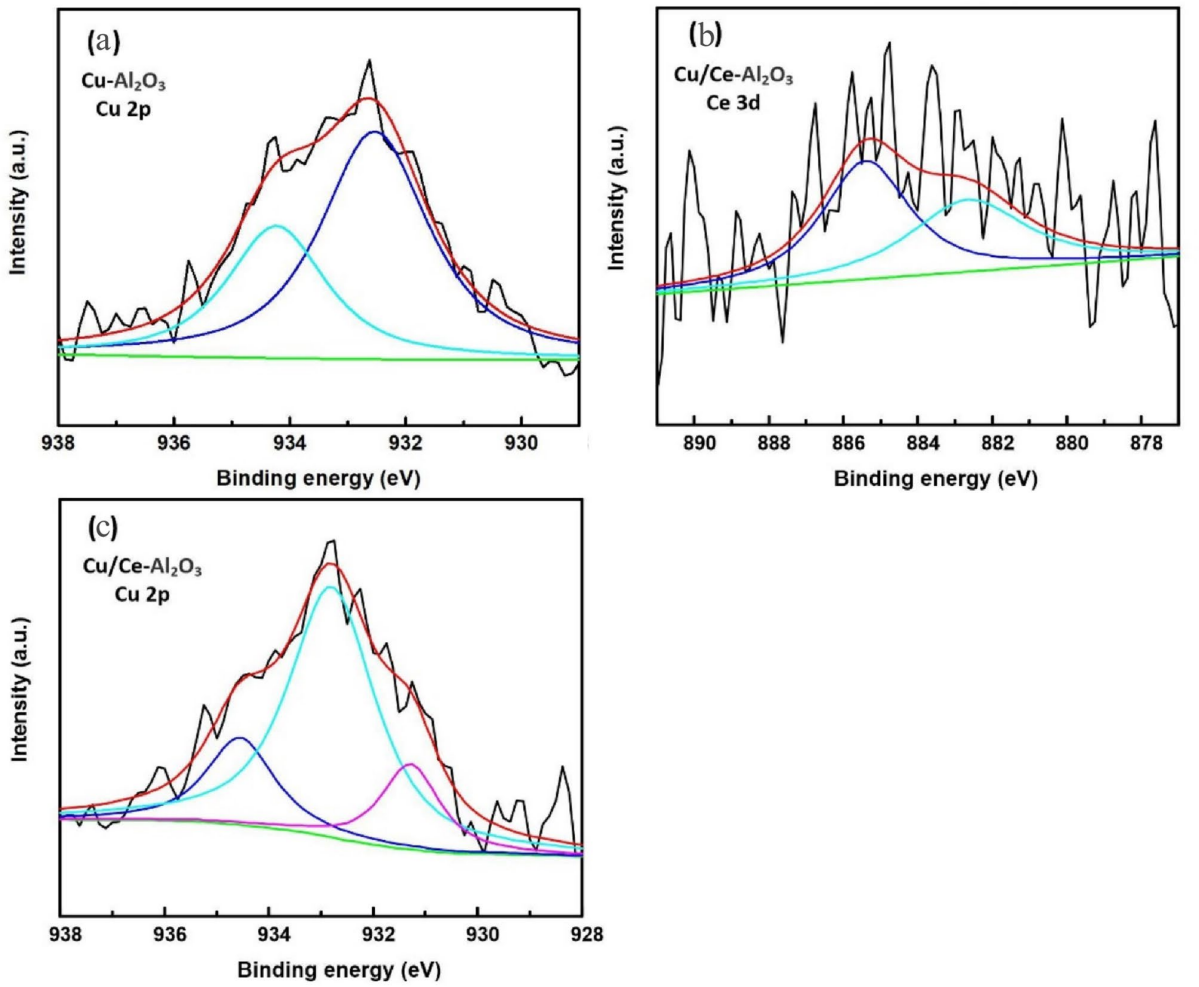
Full-range XPS spectra of catalysts: (**a**) Cu–Al_2_O_3_ Cu 2p, (**b**) Cu/Ce–Al_2_O_3_ Cu 2p and (**c**) Cu/Ce–Al_2_O_3_ Ce 3d.

XRD was used to explore the surface characteristics of the samples. It can be seen in Fig. S2 that Al_2_O_3_ and its metal-loaded catalysts presented standard peaks of pure γ-Al_2_O_3_, which are marked in Fig. S3(a) (compared to the alumina contrast card PDF#04-0875). No notable XRD diffraction peaks of the loaded metal oxide could be observed in these samples. The BET surface area and Average pore diameter of Al_2_O_3_ were bigger than Cu/Ce–Al_2_O_3_ which could indicate that the oxides of Cu and Ce attach to the surface or the pore of Al_2_O_3_. XPS was used to further investigate the surface composition and valence state of the catalysts. The atomic concentrations of the catalysts determined by XPS are listed in Table S3. In Fig. 1a,b, the binding energies at approximately 928–938 eV corresponded to Cu 2p3/2. The peaks at 934.3 and 934.6 eV were attributed to Cu^2+^ oxides (CuO)^22,23^, and the peak at 932.8 eV was related to Cu^+^ oxides (Cu_2_O)^24^. The peak at 931.3 eV in Fig. 1b was most likely due to the interaction between Cu and Ce. In Fig. 1c, the binding energies at approximately 875–895 eV corresponded to Ce 3d5/2. The peak at 885.4 eV was attributed to Ce^3+^ oxides (Ce_2_O_3_ or CeOOH)^25,26^, and the peak at 882.5 eV was related to Ce^4+^ oxide (CeO_2_). The ratios of Ce^3+^ were approximately double the ratios of Ce^4+^. A large fraction of Ce(III) has previously been found in mesoporous Ce–Ti–Zr ternary oxide millispheres, with active sites achieving a high degree of mineralization of oxalic acid (OA)^27^.

### Preparation optimization of ceramsite catalyst

**Figure 2:**
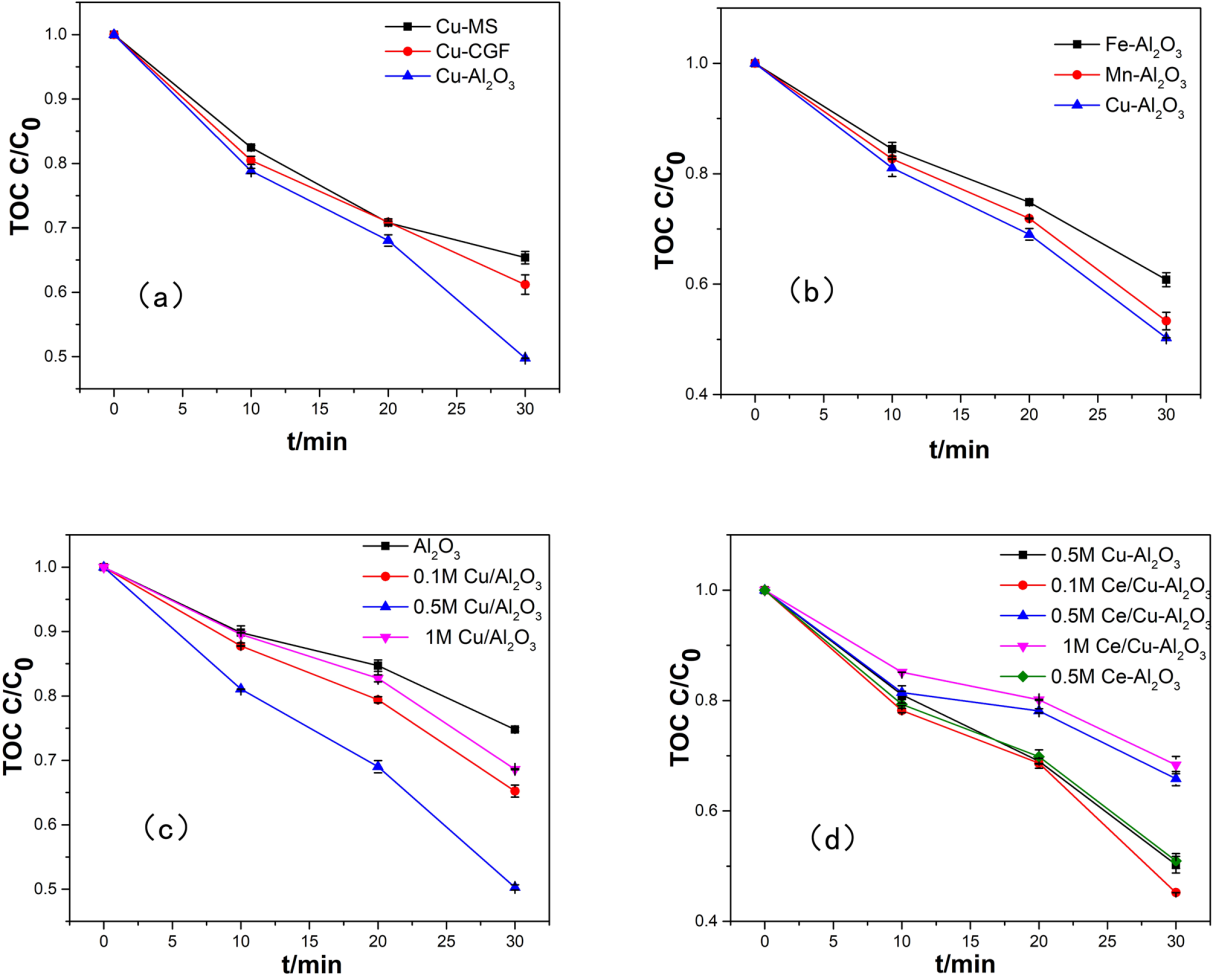
Effects of (**a**) catalyst support materials, (**b**) active components, (**c**) metal content and (**d**) metal compound on catalytic activity (experimental conditions: pH_0_, 6.49; catalyst dosage, 5.0 g/L; gas flow rate, 400 mL/min; ozone concentration, 2.90 mg/L; reaction volume, 1.0 L; and reaction time, 30 min).

To study the effect of the support materials, three copper catalysts were prepared. The immersion concentration of the copper solution was 0.5 M. The effect of the catalyst support materials on the removal efficiencies of TOC is shown in Fig. 2a. Cu–Al_2_O_3_ had the highest TOC removal rate of HA at 30 min, namely, 49.72%. The TOC removal rates using Cu-CGF and Cu-MS as catalysts at 30 min were 34.63 and 38.82%, respectively. The TOC values are very similar until 20 min when comparing the three catalysts which indicated that three catalysts have similar adsorption capacity at the beginning of the reaction. However, the adsorption capacity of Cu–Al_2_O_3_ was better than Cu-CGF and Cu-MS according to the TOC removal rate at 30 min.

From the characterization of the three support materials, we can see that the molecular sieve (MS) exhibited the largest specific surface area and metal loading rate, while its TOC removal was the lowest. It seems that the specific surface area and metal loading rate of the catalysts was not the decisive factor affecting the TOC removal rate in our study. As we know, HA shows weak acidity in solution. The chemical properties of HA are influenced by its carboxyl and hydroxyl groups. The H^+^ contained in these groups is ionized in solution, making it negatively charged under our pH conditions (6.49). Meanwhile, the pH influences the charge property of surface hydroxyl groups at the catalyst/solution interface.

1 $${\text{MeOH}} + {\text{H}}^{ + } \Leftrightarrow {\text{MeOH}}^{2 + } \quad \left( {{\text{pH}} < {\text{pHpzc}}} \right)$$

2 $${\text{MeOH}} + {\text{OH}}^{ - } \Leftrightarrow {\text{MeO}}^{ - } + {\text{H}}_{2} {\text{O}}\quad \left( {{\text{pH}} > {\text{pHpzc}}} \right)$$

The pH_PZC_ of Cu-Al_2_O_3,_ Cu-CGF and Cu-MS were 9.9, 2.6 and 2.2, respectively. At pH 6.49, the catalyst surface of Al_2_O_3_ was positively charged, while the catalyst surfaces of CGF and MS were negatively charged. The higher pH_PZC_ of Al_2_O_3_ enabled the easy adsorption of HA through electrostatic interactions. In contrast, the negative charge on the surface of GCF and MS hindered the adsorption of HA, thus affecting the removal rate of TOC. The pH_PZC_ of catalysts greatly affects the density of surface hydroxyl groups on the catalytic surface; the groups function as active surface sites, which are believed to be key to generating ·OH by ozone decomposition^28^. As we can see, the pH_PZC_ of catalysts may be the main factor affecting the TOC removal rate of HA through electrostatic adsorption of HA and acceleration of the ozone decomposition into ·OH.

The effects of different active components on the catalytic activity were studied, and the results are shown in Fig. 2b. Al_2_O_3_ was used as the catalytic carrier. The immersion concentration of the metal nitrate solution is 0.5 M. It can be observed that Cu-Al_2_O_3_ achieved the highest TOC removal rate, namely, 49.72%. The TOC removal rates of Fe–Al_2_O_3_ and Mn–Al_2_O_3_ at 30 min were 39.19 and 46.68%, respectively. As the metal wt% of Cu, Fe and Mn were 2.15, 3.67 and 1.27, respectively, so the reaction constants per mass of active metal were 0.77 mol/(L min), 0.36 mol/(L min) and 1.23 mol/(L min).

There was a close relationship between the catalytic activity and type of loaded metallic oxide. As an effective active component, the metallic oxide could enhance the generation rate of hydroxyl radicals via heterogeneous catalytic ozonation^29^. It has been reported that the surface hydroxyl groups and isoelectric points could be changed by loading different metallic oxides^18^. We can explain the results of our experiment via the isoelectric point^30^. The isoelectric points of Cu–Al_2_O_3_, Fe–Al_2_O_3_ and Mn–Al_2_O_3_ were 9.9, 9.7 and 9.8, respectively. The isoelectric points of Cu–Al_2_O_3_ and Mn–Al_2_O_3_ were very close which could explain the similar TOC removal rate at 30 min. Under neutral pH conditions, the load of metal oxide helped to change the surface charge to a positive charge and improved the utilization rate of ozone.

The influence of the metal content and metal compound on the catalytic activity was also studied. Figure 2c,d shows the effects of the metal content and metal compound on the catalytic activity of Al_2_O_3_ for the ozonation of HA. As far as catalytic ozonation was concerned, the metal oxides could function as active surface sites and increase the production rate of hydroxyl radicals. The active surface sites of the catalyst increased with increasing metal content. However, excess metal species would block channels and decrease the catalysts surface area, which could be observed from the change in specific surface area before and after metal loading (Table S2 and Table 1)^31^. Therefore, the ability of catalysts to adsorb ozone and organic pollutants in solution decreased. Meanwhile, the addition of cerium could not only increase the catalytic activity but also generate oxygen vacancies to enhance the absorption and oxidation of organic compounds. Moreover, the high activity of Cu/Ce–Al_2_O_3_ was related to the electron transport between copper and cerium. As shown in Fig. 1b,c and Fig. S4, due to the high content of Ce^3+^ (932.8 eV), the Cu/Ce–Al_2_O_3_ catalysts followed the redox equilibrium of Cu^2+^ + Ce^3+^ ↔ Cu^+^  + Ce^4+^ and shifted to the right to form more stable Cu^+^ species (885.4 eV).

### ***Influencing factors and mechanisms of catalytic ozonation of HA by Cu/Ce***–***Al***_***2***_***O***_***3***_

#### Mechanism of catalytic ozonation of HA

**Figure 3:**
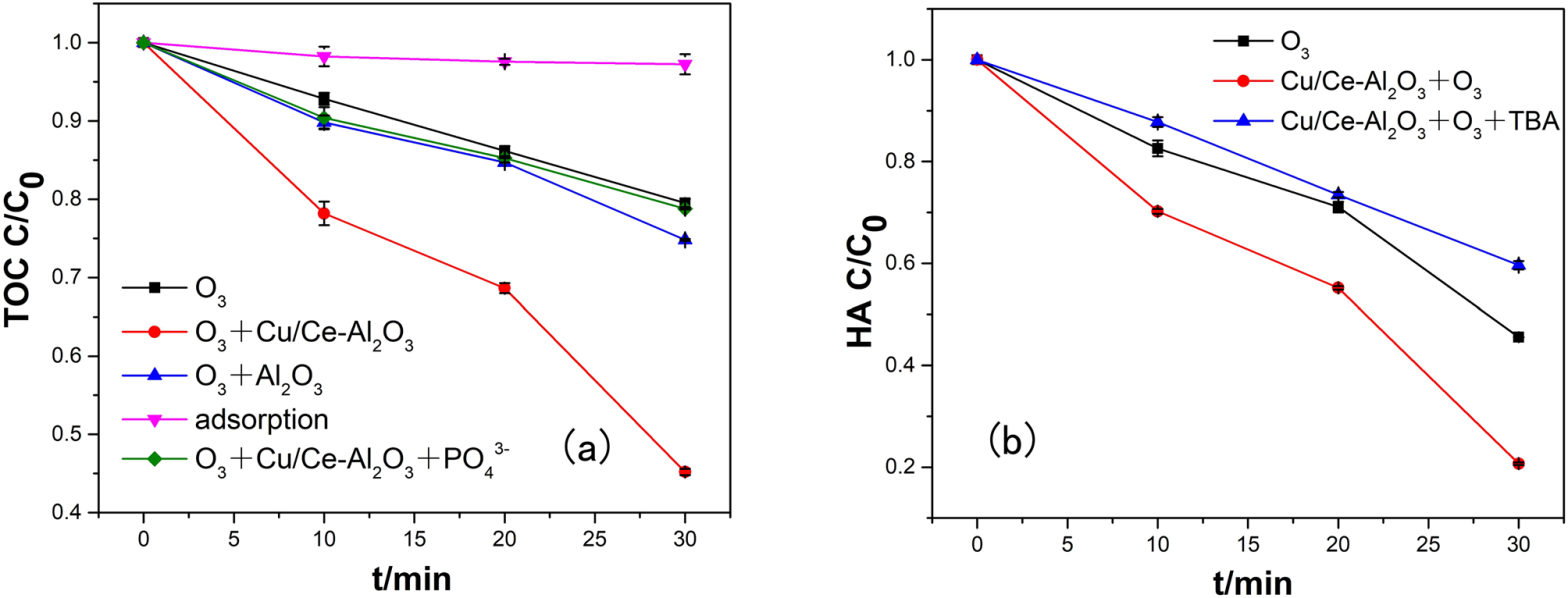
Comparison of HA removal rate among different processes. In (**a**) the square represents the simple ozone reaction, the upper triangle the ozone reaction with the addition of Al_2_O_3_, the circle represents represents the ozone reaction with the addition of Cu/Ce–Al_2_O_3_ catalyst, the lower triangle represents the adsorption reaction with the addition of Cu/Ce–Al_2_O_3_ catalyst, and the diamond represents the ozone reaction with the addition of Cu/Ce–Al_2_O_3_ catalyst and phosphate; In (**b**) the square represents the simple ozone reaction, the circle represents the ozone reaction with Cu/Ce–Al_2_O_3_ catalyst , the upper triangle represents the ozone reaction with Cu/Ce–Al_2_O_3_ catalyst and TBA (experimental conditions: pH_0_, 6.49; catalyst dosage (if used), 5.0 g/L; gas flow rate, 400 mL/min; ozone concentration (if used), 2.90 mg/L; reaction volume, 1.0 L; reaction time, 30 min; sodium phosphate dosage, 0.4 μmol/L; and tert-butyl alcohol dosage, 10 mmol/L).

The removal of HA by sole ozonation, catalytic ozonation, catalyst adsorption and ozonation with alumina were studied. Figure 3 depicts the HA mineralization efficiency among the different oxidation and adsorption processes. The adsorption of HA by Al_2_O_3_ was 2.76% in equilibrium. The ozonation of HA led to a 20.49% TOC removal level at 30 min. The simultaneous use of ozone and pure Al_2_O_3_ slightly improved the TOC removal rate at 30 min to 25.20% because Al_2_O_3_ was not the main catalytic site but functioned more as a carrier. However, in the Cu/Ce–Al_2_O_3_/O_3_ process, a remarkable improvement in the TOC removal rate was achieved. The TOC removal rate increased from 20.49% by sole ozonation to 54.79% at 30 min. It can be seen that Cu/Ce–Al_2_O_3_ had a significant effect on removal of HA.

The pH_PZC_ of a catalyst is known to greatly affect the density of surface hydroxyl groups on the catalytic surface^18^. From our experimental data, the Cu/Ce–Al_2_O_3_ catalysts with the highest electric point produced the greatest catalytic effect, which is consistent with previous conclusions on the relationship between the isoelectric point, presence of surface hydroxyl groups and catalytic effect^18^. To study the influence of the surface hydroxyl groups during the Cu/Ce–Al_2_O_3_/O_3_ process, phosphate radicals were added to the ozonation system. Phosphate, known as a strong Lewis base, can be strongly bonded with surface Lewis acid sites of the catalyst, thereby inhibiting the adsorption of O_3_ and water onto catalysts^32,33^. TOC removal was greatly inhibited in the presence of 0.4 μM phosphate, leading to a 21.21% yield that was similar to that achieved by ozonation alone. This result indicated that the surface hydroxyl group played an important role in the catalytic behavior by adsorbing O_3_ and water molecules on Lewis acid sites and generating hydroxyl radicals on the surface. Meanwhile, the ·OH radical was indispensable to the reaction of HA with Cu/Ce–Al_2_O_3_/O_3_ from Fig. 3b (The discussion of ·OH generation can be found in supporting information). The metallic oxide plays an important role for production of hydroxyl radical from ozone which involves a series of chain reactions by electron transfer from a metal ion with variable valence to ozone. Elimination of the HA (P) by ozone and ·OH can be represented by the following simple reactions:

3 $${\text{O}}_{3} + {\text{P}}\to ^{{{\text{K}}_{{{\text{O}}_{3} }} }} {\text{M}}$$

4 $$\cdot{\text{OH}} + {\text{P}}\to ^{{{\text{K}}_{{{\text{OH}}}} }} {\text{M}}$$

Therefore, the overall removal of M can be described by combining two second-order rate equations:

5 $$- \frac{{{\text{d}}\left[ {\text{M}} \right]}}{{{\text{d}}_{{\text{t}}} }} = {\text{k}}_{{{\text{O}}_{3} }} \left[ {{\text{O}}_{3} } \right]\left[ {\text{P}} \right] + {\text{k}}_{{{\text{OH}}}} \left[ {^{ \cdot } {\text{OH}}} \right]\left[ {\text{P}} \right]$$

R_ct_ is assumed to be a constant value for an ozonation process E, reflecting the catalytic activity:

6 $${\text{R}}_{{{\text{ct}}}} = \frac{{\left[ {^{ \cdot } {\text{OH}}} \right]}}{{\left[ {{\text{O}}_{3} } \right]}}$$

Substituting Eq. (6) into Eq. (7) and integrating, the rate equation describing the removal of P becomes:

7 $$\ln (\frac{{\left[ {\text{P}} \right]}}{{\left[ {\text{P}} \right]_{0} }}) = - \left( {{\text{k}}_{{{\text{O}}_{3} }} + {\text{k}}_{{{\text{OH}}}} {\text{R}}_{{{\text{ct}}}} } \right)\smallint \left[ {{\text{O}}_{3} } \right]{\text{d}}_{{\text{t}}}$$

The R_ct_ value of Cu/Ce–Al_2_O_3_ is about four times than what of sole ozone according to the Eq. (7), which indicating the excellent ozone efficiency of the catalyst.

#### Effect of the ozone concentration of the aqueous solution on the catalytic ozonation by Cu/Ce–Al_2_O_3_

**Figure 4:**
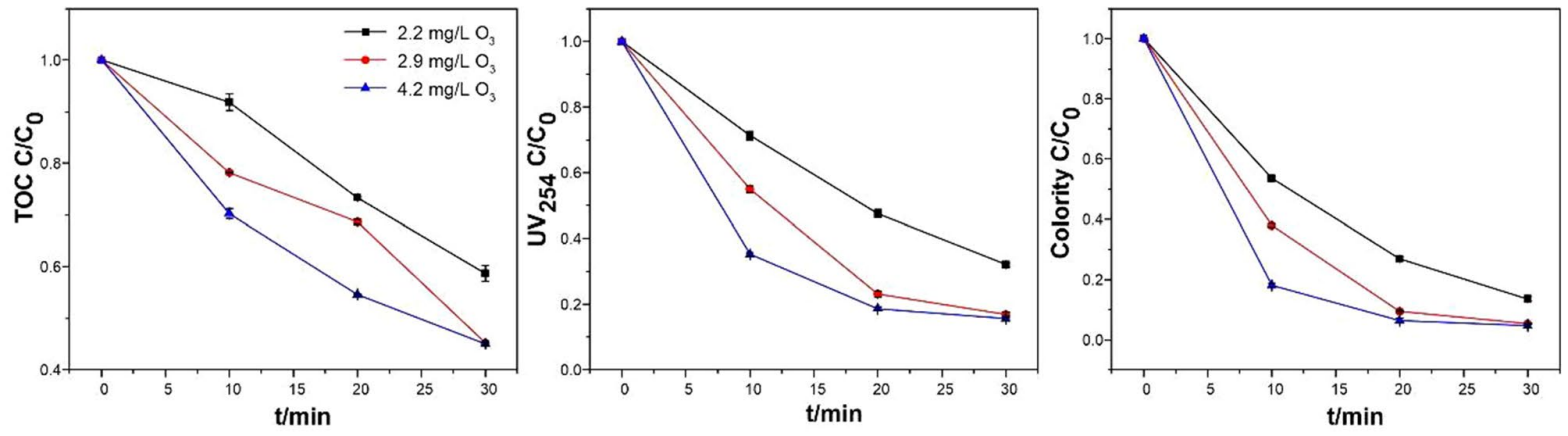
Effect of ozone dosage in an aqueous solution on catalytic ozonation by Cu/Ce–Al_2_O_3_ (experimental conditions: pH_0_, 6.49; catalyst dosage, 5.0 g/L; gas flow, 400 mL/L; reaction volume, 1.0 L; and reaction time, 30 min).

The ozone concentration was one of the factors that affected the catalytic effect. Increasing the concentration of ozone in water could accelerate the ozone decomposition efficiency and increase the rate of hydroxyl radical formation in the reaction system. UV_254_ can represent the content of aromatic structures and double bonds of organic pollutants^34^. Furthermore, UV_254_ is also a method for characterizing HA. HA is also a major substance that causes the chroma of water, so chroma was also used to characterize the removal of HA. The effects of ozone concentration on removal efficiencies of TOC, UV_254_ and chroma are shown in Fig. 4. As shown in Fig. 4, when the ozone concentration increased from 2.2 to 2.9 mg/L, the TOC removal efficiency increased from 41.30 to 54.79%, the UV_254_ removal efficiency increased from 67.91 to 83.21%, and the chroma removal efficiency increased from 86.45 to 94.72% at an oxidation time of 30 min. However, when the ozone concentration was gradually increased to 4.2 mg/L, the removal rate of HA changed very little. Because the number of active surface sites of catalysts was limited, the utilization of ozone reached saturation. An excess of ozone could not increase the amount of active substances produced by the catalysts. Ozone and hydroxyl radicals could destroy the aromatic structures and double bonds, which caused a significant decrease in the UV_254_ value. Meanwhile, the chroma was largely the result of chromophores (e.g., C=O, C=C, etc.) and auxiliaries (e.g., –NH_2_, –OH, etc.). Ozone could easily destroy unsaturated groups, thereby improving the chroma removal rate^35^.

A comparison of the DOC removal efficiencies achieved in this study with literature data for catalytic ozonation processes is given in Table 2^36–42^. In this study, a 54.79% TOC and an 83.21% UV_254_ removal rate were achieved for the Cu/Ce–Al_2_O_3_/O_3_ process. Considering the simplicity of the catalyst preparation method and availability of alumina support material, Cu/Ce–Al_2_O_3_ achieved a suitable removal of HA.

**Table 2:** Comparison of DOC removal efficiencies of catalytic ozonation processes.

Types of catalysts	Humic acid concentration	Catalyst dosage	Ozone dosage	Reaction time (min)	Removal efficiency (%)	References
FeOOH	DOC:3.5 mg/L	5 mg/L	10 mg/L	40	54	Park et al.^36^
AC	30 mg/L	1 g/L	40 mg/min	120	33.30	Kawasaki et al.^37^
Zeolite	DOC:7.05 mg/L	25 g/L	19.2 mg/min	30	45	Wang et al.^38^
Zeolite/TiO_2_	DOC:7.05 mg/L	25 g/L	19.2 mg/min	30	65	Lee et al.^39^
Fe/MgO	50 mg/L	0.5 g/L	40 mg/min	30	50	Lee et al.^39^
TiO_2_	DOC:9.85 mg/L	1 mg/L	10 mg/L	40	54	Molnar et al.^40^
Pumice	30 mg/L	0.45 mg/min	10 mg/L	30	UV_254_:90	Asgari et al.^41^
ICZ	30 mg/L	0.75 mg/L	10 mg/L	60	62.01	Gumus et al.^42^
GAC	30 mg/L	0.75 mg/L	10 mg/L	60	48.08	Gumus et al.^42^
Cu/Ce–Al_2_O_3_	50 mg/L	5 g/L	2.2 mg/L	30	54.79	This study

#### Effect of the initial pH on the catalytic ozonation of HA

**Figure 5:**
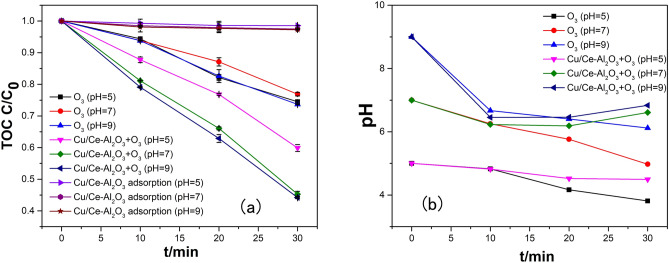
Effect of the initial pH on the catalytic ozonation of HA (**a**) and changes in the pH among the different process (**b**) (experimental conditions: catalyst dosage, 5.0 g/L; gas flow rate, 400 mL/min; ozone concentration, 2.90 mg/L; reaction volume, 1.0 L; and reaction time, 30 min).

The initial pH of the solution was an important factor affecting the catalytic activity of the catalyst. The pH can directly affect the efficiency of ozone decomposition, the form of organic pollutants and the surface properties of catalysts^29,43^. The influence of initial pH on the catalytic ozonation of HA was investigated for the different processes, including sole ozonation, catalytic ozonation and adsorption of HA by Cu/Ce–Al_2_O_3_. As shown in Fig. 5a, a TOC removal rate of approximately 25% via sole ozonation was obtained under different pH conditions, and the removal rate was slightly lower under neutral conditions. The latter was because alkaline conditions were more conducive to the production of hydroxyl radicals, and the dissociation degree of HA was higher under acidic conditions. However, for the Cu/Ce–Al_2_O_3_/O_3_ process, the removal efficiency of TOC under acidic conditions was significantly inhibited and was 15% lower than under neutral and alkaline conditions. Meanwhile, the adsorption capacity of the catalyst under acidic conditions was also slightly lower than under neutral and alkaline conditions. For catalytic ozonation, the surface charge properties of the catalysts played an important role in O_3_ decomposition and ·OH generation. Catalysts covered by surface hydroxyl groups would be protonated or deprotonated when the pH of the solution was lower than or higher than pH_PZC_. Several studies have verified that when the surface is near electric neutrality and protonated, the surface has a strong reactivity toward ozone^44,45^. In our study, the pH_PZC_ of Cu/Ce–Al_2_O_3_ was 10.2, and pH values of 7 and 9 were closer to the isoelectric point. Meanwhile, the surface hydroxyl groups were protonated under our pH conditions. When the solution was weakly acidic (pH = 5), the surface of HA was positively charged, and electrostatically repulsed the groups on the catalyst surface and decreased the reaction rate. The pH changes are shown in Fig. 5b. After reaction, the pH of all experimental groups decreased which could be due to the generation of acid intermediates. The metals leaching can also be seen in Table S4.

### ***Characteristics of the organic compounds before and after the Cu/Ce***–***Al***_***2***_***O***_***3***_*** reaction***

**Figure 6:**
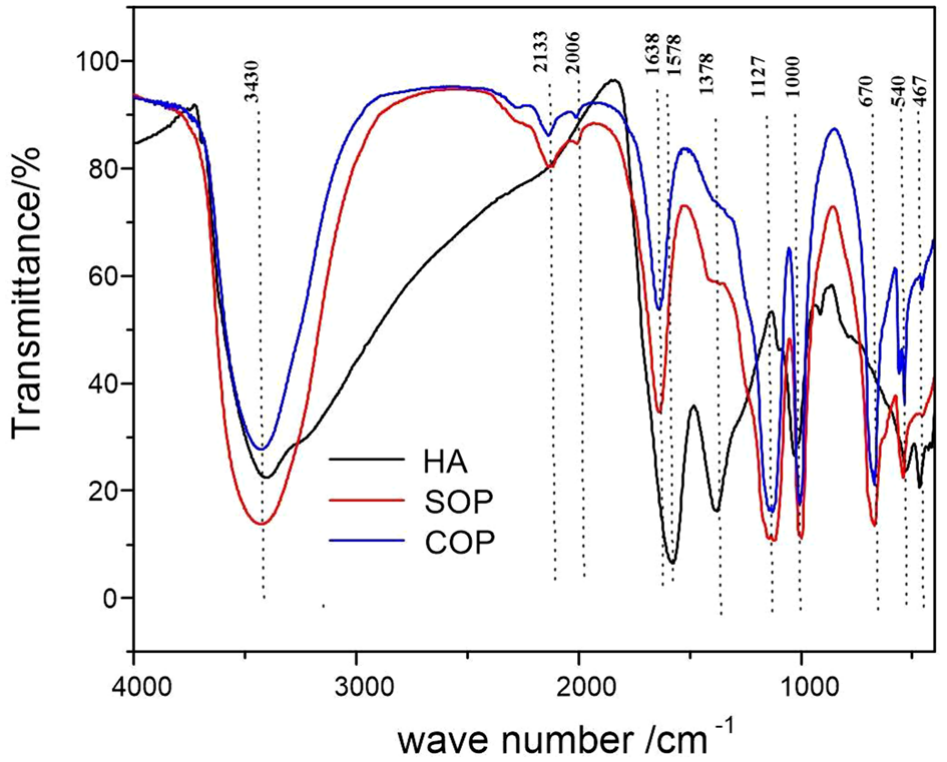
FT-IR spectra of dried humic acid (HA), catalytic ozonation (COP) and sole ozonation process (SOP) samples.

Infrared (IR) spectroscopy was used to identify the functional groups with characteristic group frequencies^46,47^. Figure 6 shows the FT-IR spectra of dried samples derived from humic acid (HA), catalytic ozonation process (COP) and sole ozonation process (SOP). The broad absorption peaks of the HA, SOP and COP samples at 3430–3400 cm^−1^ were the stretching vibration absorption of –OH (3700–3200 cm^−1^, respectively) or –NH_2_ (3500–3300 cm^−1^). The –OH and –NH_2_ were auxochrome groups, which contributed to the formation of chroma. The SOP and COP samples had weak absorption peaks (2133 cm^−1^ and 2006 cm^−1^, respectively) at 2500–2000 cm^−1^, which were mainly caused by the asymmetric stretching vibration of conjugated double bonds, indicating that unsaturated double bonds still existed in the treated samples. In the functional regions (1900–1500 cm^−1^), HA had an absorption peak at 1578 cm^−1^, representing the aromatic structure C=C (1620–1450 cm^−1^). The SOP and COP samples had an absorption peak at 1638 cm^−1^, representing C=O (1870–1600 cm^−1^) or C=C in aldehydes and ketones (1680–1620 cm^−1^). These results indicated that HA itself contained additional aromatic structures. After SOP and COP treatment, the aromatic structures were destroyed, and C=O or C=C was formed, which resulted in the decrease in the UV_254_ (see Fig. 4). The peak strength after the COP treatment was much weaker than that after the SOP treatment, indicating that the COP treatment was more thorough. There were additional absorption peaks in the fingerprint area (1500–600 cm^−1^). Further analysis results are provided in supporting information.

A total of 15 organic species were detected by GC–MS in the HA sample, including alkanes, phenols, acids, amides, and olefins. After the COP treatment, the content of 13 species decreased, 2 species were completely removed and 3 species were newly generated. The sum of the peak areas of the organic compounds was reduced by 59.42%, to a level 6.54% less than that of the SOP treatment, indicating that HA could be effectively degraded during the COP treatment. The main organic species before and after COP and SOP treatment that were detected with relative peak areas > 2% are listed in Table S5. The reduction in UV_254_ and chroma were most likely a result of the partial degradation of organics with double bonds and benzene rings, such as 1-Docosene, Phenol,2,4-di-t-butyl-6-nitro-, trans-13-Docosenamide, Cyclopentene,1,2,3,3,4-pentamethyl-. The main organic substances after the COP treatment were long chain oxygen-containing aliphatic compounds.

### Catalytic stability and toxicity assessment

To examine the catalytic stability of the catalysts, Cu/Ce–Al_2_O_3_ was recycled three times in the catalytic ozonation experiments. As shown in Table 3, the TOC removal rate was slightly reduced at cycles 2 and 3, and the leaching of the metal content was relatively low, which was approximately 1% of the total metal loading. Also, the structure of the alumina balls was not affected by the ozonation catalytic process. So after three recycles, the Cu/Ce–Al_2_O_3_ catalyst remained stable in the catalytic ozonation of HA.

**Table 3:** Effect of the number of cycles on TOC removal and leaching of the metal content in the Cu/Ce–Al_2_O_3_/O_3_ process.

Cycle times	1	2	3
TOC removal rate (%)	54.79	51.02	48.97
c(Cu) (μg/L)	472	302	328
c(Ce) (μg/L)	49.9	28.2	34.1

Measurement of acute toxicity is also an important factor in evaluating the effect of catalytic ozonation, especially since the intermediate products of the ozonation process may be more toxic than the original organic substances^48^. In this section, the acute toxicity to luminescent bacteria of humic acid(HA), catalytic ozonation process (COP), sole ozonation process (SOP) and 0.5 mg/L Cu^2+^ treatment water samples were tested. The results are shown in Table 4.

**Table 4:** Ecotoxicity analysis of catalysts.

Samples	HA	SOP	COP	0.5 mg/L Cu^2+^
EC_HgCl_^a^ (mg/L)	< 0.02	0.18	0.08	0.08
Relative luminosity (%)	86.5	14.5	53.6	53.6

^a^EC_HgCl_: Equivalent concentration of mercuric chloride.

As can be seen in Table 4, the acute toxicity of HA raw water was low, and the toxicity increased significantly after SOP, indicating that toxic intermediates may be produced. The toxicity of COP was 56% lower than that of SOP, indicating that toxic intermediates were destroyed. The leaching of copper from the catalyst may also affect toxicity, so HA solution spiked with 0.5 mg/L copper was also studied. The 0.5 mg/L copper ion control group was found to have the same toxicity as COP, indicating that the COP process promoted by metal catalysts will weaken the toxicity compared with SOP if the release of metal irons could be avoided. So it is important to reduce or avoid the leaching of copper, for example by enhancing the load of the organometallic precursor. The main toxic and refractory organics in wastewater include phenolic compounds, polynuclear aromatic hydrocarbons (PAH), nitrogenous heterocyclic compounds (NHC), long-chain hydrocarbons, ammonia, cyanide, etc.^49^. The toxicity change may be related to the change of toxic and refractory compounds in wastewater. Combined with the GC–MS analysis of HA before and after treatment, 1-nonadecene, pentanoic acid, 2,2,4-trimethyl-3-carboxyisopropyl, isobutyl ester, phenol, 2,4-bis(1,1-dimethylethyl)-, hexadecane, 2,6,10-trimethyl-, octadecanoic acid, nonadecane were intermediates produced by COP and SOP. These substances are all phenolic compounds or long-chain hydrocarbon compounds, so they may be potential toxic substances that cause increased toxicity after treatment. At the same time, after COP, hexadecane, 2,6,10-trimethyl, octadecanoic acid, nonadecane are completely removed, and the peak area of pentanoic acid, 2,2,4-trimethyl-3-carboxyisopropyl, isobutyl ester, phenol, 2,4-bis(1,1-dimethylethyl)- was lower than that of SOP. It showed that COP could effectively degrade toxic intermediates and reduce the acute toxicity generated during the treatment by generating a high oxidation active hydroxyl radical.

### The degradation efficiency of HA by catalytic ozonation in real wastewater

**Figure 7:**
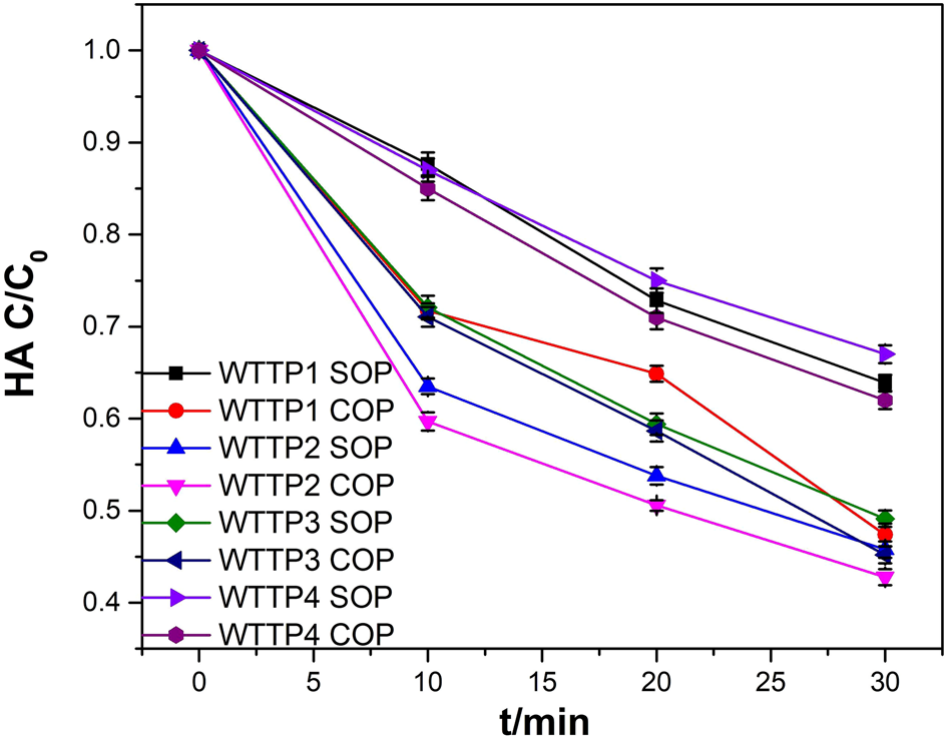
HA removal in four samples of biochemical effluent wastewater (experimental conditions: unadjusted pH_0_; catalyst dosage (if used), 5.0 g/L; gas flow rate, 400 mL/min; ozone concentration (if used), 2.90 mg/L; reaction volume, 1.0 L; reaction time, 30 min).

Four samples from secondary sedimentation effluents from four WTTPs were used to examine the degradation efficiency of HA by catalytic ozonation in real wastewater. Figure 7 shows HA removal in four samples of biochemical effluent wastewater.

**Figure 8:**
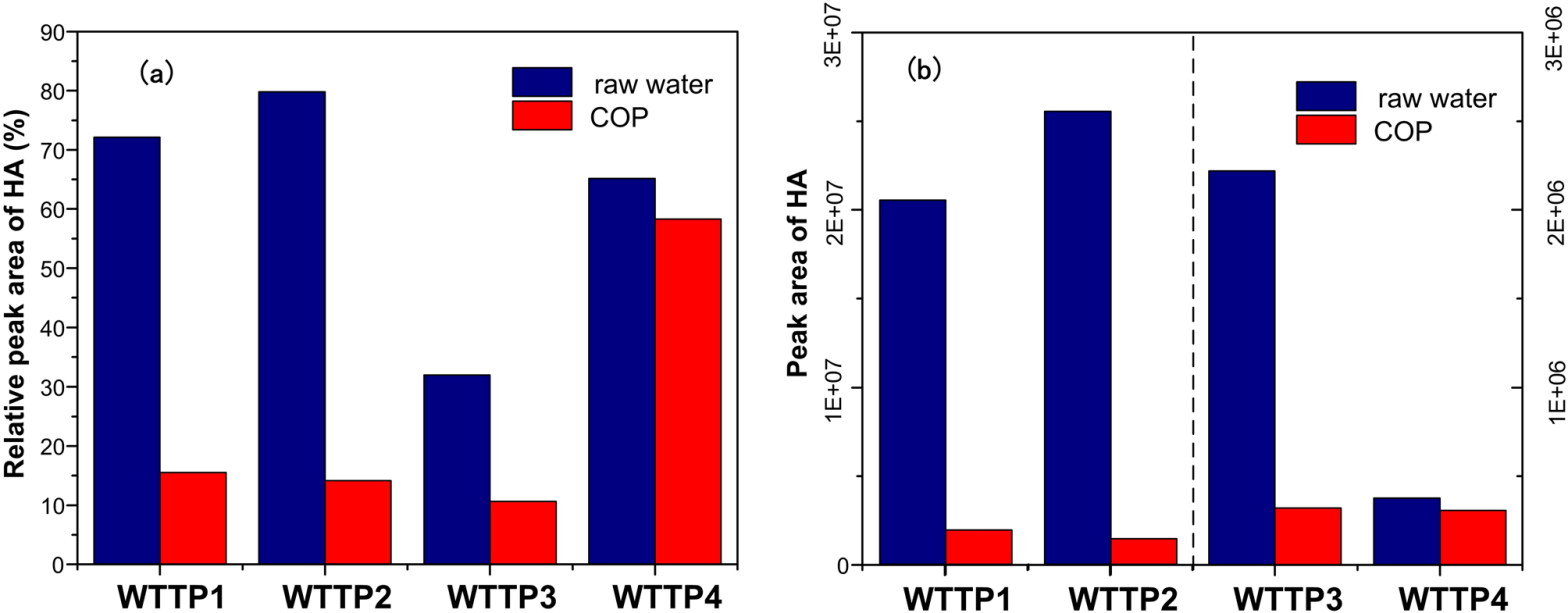
Changes of (**a**) relative peak area and (**b**) peak area of HA before and after COP treatment.

As can be seen from Fig. 7, the HA removal rates by COP in four samples of biochemical effluent were 52.62%, 57.20%, 54.80% and 38%, respectively, which was 16.52%, 2.99%, 3.93% and 6% better than with ozone oxidation alone. The improvements in HA removal efficiency in real wastewater were lower than in simulated wastewater, possibly because the types of HA in real wastewater are different. The results of GC–MS analysis of humic acid substances in four samples of biochemical effluent wastewater are summarized in Table S6–S9. Figure 8 shows the changes of relative peak area and peak area of HA before and after COP treatment.

It can be seen from Fig. 8 that the proportion of humic acid substances in four samples of biochemical effluent wastewater was decreased after COP. Compared with the raw effluent, the humic acid substances peak area decreased by 90.59%, 94.33%, 85.54% and 18.72% in the four samples. In WTTP1 and WTTP2 effluent, where phenol, 2,4-bis(1,1-dimethylethyl)- was initially high, the removal efficiency was significant. The high proportions of 9-octadecenamide, (z)-, n-hexadecanoic acid and phenol, 2,4-bis(1,1-dimethylethyl)- in WTTP3 effluent were completely removed after COP. Overall, the removal efficiencies of humic acid substances in WTTP1, WTTP2 and WTTP3 wastewater by COP were high, all of which were more than 80%. The main reason was that the reaction rates of phenol and its derivatives with ozone and hydroxyl radicals are 1.8 × 10^6^ M^−1^ s^−1^ and 6.1 × 10^9^ M^−1^ s^−1^, respectively. Most of the phenolic compounds were oxidized rapidly and further removed by the hydroxyl radicals produced by catalytic ozonation. The remaining parts were converted to dimethyl phthalate and phenoxyl compounds^50^. Humic acid substances such as hexadecane, 2,6,10,14-tetramethyl-, erucic acid, pentadecane, 2,6,10-trimethyl- in WTTP4 effluent had a low removal rate after COP, hence overall low removal efficiency of humic acid substances in WTTP4 effluent. This was mainly due to the fact that these kinds of humic acid substances are long-chain aliphatic compounds, which are very stable and are difficult to remove by chemical oxidation without high temperature^51^. The removal efficiency of humic acid substances by SOP was in all cases lower than that by COP.

Acute toxicity of wastewater is also an important factor in evaluating the effect of catalytic ozonation on wastewater treatment. Compared with the potential toxic substances in the simulated HA experiment, WTTP1 and WTTP2 wastewater contained phenol,2,4-bis(1,1-dimethylethyl)-; WTTP3 wastewater contained phenol, 2,4-bis(1,1-dimethylethyl)-, octadecanoic acid, nonadecane; WTTP4 wastewater contained hexadecane, 2,6,10-trimethyl-. After COP, phenol, 2,4-bis(1,1-dimethylethyl)-, octadecanoic acid in WTTP3 wastewater were completely removed, phenol,2,4-bis(1,1-dimethylethyl)- in WTTP1 and WTTP2 wastewater was greatly removed, and nonadecane in WTTP3 wastewater increased slightly. Octadecanoic acid was formed in WTTP2 wastewater by SOP. The results showed that potential toxic humic acid substances in real wastewater were effectively removed by COP.

**Figure 9:**
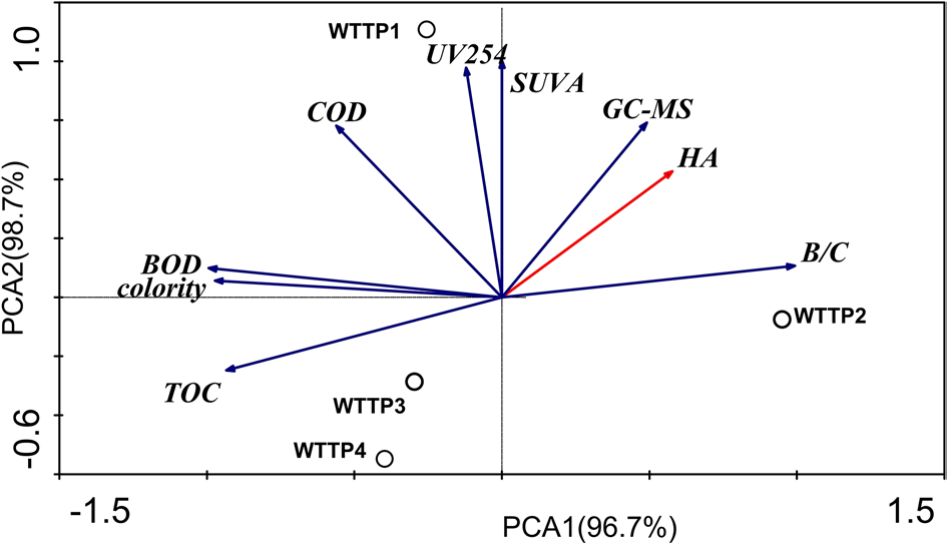
PCA analysis of correlation between humic acid and other indicators.

The removal efficiency of basic water quality indicators and HA characterization indexes by catalytic ozonation were studied. The results are summarized in Table S10. In order to explore the correlation between the removal efficiency of humic acid by catalytic ozonation and other indicators in wastewater, the removal rate of HA, TOC, chroma, UV_254_, COD, GC–MS humic acid substances, SUVA, BOD and the increase rate of B/C were chosen as variables, and analyzed by principal component analysis (PCA). The results are shown in Fig. 9.

It can be seen from Fig. 9 that PCA1 and PCA2 account for 96.7% and 98.7% of the total difference, respectively. The four wastewaters are divided into three regions: WTTP1 was located in the second quadrant, WTTP2 was located in the fourth quadrant, WTTP3 and WTTP4 were located in the third quadrant, indicating that the water quality indicators of different effluent samples were significantly different and had good representativeness. Meanwhile, it was found that HA had a significant positive correlation with B/C of basic water quality index, indicating that B/C (biodegradability) increased with the removal of HA; the correlation between HA and COD and TOC was not obvious. Most of the HA removal process was not completely mineralized, but rather produced some biodegradable intermediates. At the same time, UV_254_, SUVA, GC–MS analysis of three indicators for characterizing HA, the correlation with HA was GC–MS > SUVA > UV_254_; GC–MS analysis and HA had significant correlation, indicating that GC–MS analysis could better reflect the removal performance of HA, which was superior to the conventional HA characterization indexes SUVA and UV_254_.

## Conclusion

Catalysts were prepared with different granular carriers (Al_2_O_3_, ceramic grain filters and molecular sieves) loaded with different active components (Cu, Fe, and Mn) to remove HA by ozonation. Cu-Al_2_O_3_ showed the highest TOC removal rate, namely, 49.72%. The high pH_PZC_ and density of surface hydroxyl groups contributed to the high efficiency of the catalysts. Further modification of the catalysts was carried out. The results showed that when 0.1 M cerium was mixed with a 0.5 M copper salt solution, the TOC removal rate increased to 54.79%. This was due to the electron transfer effect of Cu^2+^/Cu^+^ and Ce^3+^/Ce^4+^ revealed by XPS.

The ozone dosage and initial pH influenced the catalytic ozonation of HA. The addition of tert-butyl alcohol (TBA) and phosphate groups in the Cu/Ce–Al_2_O_3_/O_3_ process caused a significant decrease in the removal efficiency, indicating that the generation of hydroxyl radicals and presence of Lewis acid sites were indispensable for the catalytic ozonation by Cu/Ce–Al_2_O_3_. FT-IR analysis showed that the chroma and UV_254_ of the catalytic reaction decreased through a change in the structure and number of functional groups or organic compounds. GC–MS analysis indicated that much of the organic matter was degraded in large quantities. The addition of catalysts could effectively degrade toxic intermediates and reduce the acute toxicity produced by ozonation. The degradation efficiency of HA by catalytic ozonation in real wastewater effluent varied according to the types of humic acid substances determined by GC–MS. The HA was largely removed and transformed into biodegradable intermediates.

In summary, this study has demonstrated that Cu/Ce–Al_2_O_3_ is an efficient, stable and practical catalyst for the degradation of HA.

## Supplementary Information

Below is the link to the electronic supplementary material.

Supplementary materials
