# Supplementary material for: Mechanism and toxicity evaluation of catalytic ozonation over Cu/Ce–Al2O3 system aiming at degradation of humic acid in real wastewater
Source: Sci Rep. 2021 Apr 22;11:8748. doi: 10.1038/s41598-021-83804-x (PMC8062596; doi:10.1038/s41598-021-83804-x)
Supplement: Supplementary file 1 — Supplementary materials [file 41598_2021_83804_MOESM1_ESM.doc]

**Supplementary materials for**

# Mechanism and toxicity evaluation of catalytic ozonation over Cu/Ce-Al_2_O_3_ system aiming at degradation of humic acid in real wastewater

Xi Tang, Yifei Zhang, Weiqi Li, Jinju Geng, Hongqiang Ren, Ke Xu*

State Key Laboratory of Pollution Control and Resource Reuse, School of the Environment, Nanjing University, Nanjing 210023, Jiangsu, PR China

*Corresponding author:

1.Ke Xu

State Key Laboratory of Pollution Control and Resource Reuse

School of the Environment, Nanjing University

N.O.163, Xianlin Avenue, Qixia District, Nanjing 210023, Jiangsu, PR China

Tel.: 86-025-89680512; fax: 86-025-89680512

E-mail address: kexu@nju.edu.cn

**List of captions:**

Text S1 Preparation and characterization methods of catalysts

Text S2 GC-MS analysis procedure

Text S3 Acute toxicity test of luminescent bacteria

Text S4 Effect of the metal content and metal compound on the catalytic activity

Text S5 Effect of the hydroxyl radical scavenger

Text S6 pH changes and metals leaching among the different process

Text S7 The analysis of FT-IR spectra in fingerprint area (1500-600 cm^-1^)

Fig. S1 Effect of catalyst dosage on catalytic ozonation of HA

Fig. S2 SEM images of Al_2_O_3_ (a), Cu-Al_2_O_3_ (b) and Cu/Ce-Al_2_O_3_ (c)

Fig. S3 XRD patterns: (a) Al_2_O_3_ catalysts, (b) GCF catalysts and (c) MS catalysts

Fig. S4 Full-range XPS spectra of catalysts: Al_2_O_3_

Table S1 Basic water quality of four kinds of biochemical tailwater

Table S2 Physical properties of the catalyst support materials

Table S3 Atomic concentrations of the catalysts by XPS

Table S4 Effect of the initial pH of the solution on the leaching of metals in the Cu/Ce-Al_2_O_3_/O_3_ process.

Table S5 The compounds identified analysis of humic acid (HA), catalytic ozonation process (COP) and sole ozonation process (SOP) samples

Table S6-S9 Analysis of Humic Acid Substances in WTTP1-WTTP4 by GC-MS

Table S10 the removal efficiency of humic acid and other indicators in wastewater by catalytic ozonation

**Text S1 Preparation and characterization methods of catalysts**

Activated Al_2_O_3_, ceramic grain filters and molecular sieves were used as catalyst support materials. They were all 3 to 5 mm diameter particle balls. The activated Al_2_O_3_ was purchased from Shanghai Macklin Reagent Co., and the ceramic grain filters and molecular sieves were purchased from the Lvxinyuan Company, China. The physical properties of the catalyst support materials are summarized in Table S1. These three materials with different physical properties were used to compare the effects of supported materials on the catalytic performance. The catalysts were prepared by the impregnation method. For cleaning of impurities and activation, the catalyst carrier was first soaked in a 1 M HCl solution for 24 h at room temperature, and then the modified carrier was immersed in a salt solution for 24 h. Subsequently, the carrier was heated in an oven at 105 °C, calcined at 500 °C for 4 h, and then cooled in the ambient environment.

The surface area and pore size distribution of the catalyst were characterized using the Brunauer-Emmet-Teller (BET) method with a surface area and pore distribution analyzer (ASAP 2020, USA). Values of the point of zero charge (pH_PZC_) of the catalysts were determined via a potentiometer (Zetasizer Nano ZS90, United Kingdom). The X-ray diffraction (XRD) data were analyzed on a diffractometer with a Cu Kα source (X’TRA, ARL, Switzerland), and X-ray photoelectron spectroscopy (XPS) data were analyzed on a spectrometer (PHI 5000 VersaProbe, Ulvac-PHI). An ICP-OES (Angilent 5100) was used to measure the metal content in the metal-based catalyst. The surface morphology and element analysis of the catalyst were observed by a scanning electron microscope equipped with an energy dispersive X-ray spectroscope (SEM-EDS, S-3400N II, Hitachi, Japan).

**Text S2 GC-MS analysis procedure**

Before GC-MS analysis, the pretreatment steps of the water sample are as follows. Uniformly mix 250 mL water sample, 2 g NaCl and 60 mL methylene chloride, then shake well and let stand for 10 min till the organic phase to separate and filter. After the filtrate was concentrated to dryness in a 45 °C water bath, the volume was adjusted to 1 mL.

The parameters of purge-and-trap parameter, gas chromatography, and mass selective detector are as follows:

**Purge-and-trap (OI, 4660) parameter:** purge temperature: room temperature, purge rate: 40 mL/min, purge time: 10 min, dry purge time: 1 min, pre-desorption temperature: 180 °C, desorption temperature: 190 °C, desorption time: 2 min, bakeout time: 6 min

**Gas chromatography parameter (Agilent 7890A**)**:** injector temperature: 220 °C, injector type: spitless (10:1), temperature programming: 35 °C (2 min) → 5 °C/min → 120 °C → 10 °C/min → 220 °C (2 min), carrier gas: helium, gas flow rate: 1.0 mL/min

**Mass selective detector parameter (Agilent** **5977B):** ion source: EI source, ion source temperature: 230 °C, ion energy: 70 eV, scanning mode: full scan, scanning range: m/z35-270amu, solvents delay: 2.0 min, electron multiplying voltage: same with tune voltage, transfer line temperature: 280 °C

**Text S3 Acute toxicity test of luminescent bacteria**

The biological toxicity of treated water samples was determined by DXY-2 biological toxicity tester and related equipment (Nanjing Institute of Soil Research, Chinese Academy of Sciences). Referring to "Water Quality-Acute Toxicity Measurement-Luminescent Bacteria Method" (GB/T15441-1995), the biological toxicity tester was turned on 15 minutes in advance and adjusted to zero for reserve. CK tubes and test tubes are arranged in sequence on the test tube rack. Each CK tube is added with 2 mL 3% NaCl solution, and each test tube is added with 2 mL sample solution, i.e. each sample solution corresponds to a blank parallel. The freeze-drying powder of Photobacterium was taken out and mixed with 1 mL cold 2.5% NaCl solution to recover the bacterial freeze-drying agent. The whole process of recovery was carried out in ice water. Accurately absorb 10 mu L resuscitating bacterial solution and add each tube one by one. When the reaction between luminescent bacteria and the sample reaches the end time, the sample is measured. Before the test, the salinity of each water sample was adjusted to 3%, and the pH was adjusted to neutral. Three parallel experiments were carried out. Regression equation of mercury chloride concentration and relative luminescence was T=84.95-391.3C, C stands for equivalent concentration of mercuric chloride(mg/L)，T stands for relative luminosity (%), and EC_50HgCl_=0.11 mg/L, r=0.952, P≤0.01。

**Text S4 Effect of the metal content and metal compound on the catalytic activity**

To study the influence of the metal content and metal compound on the catalytic activity, Al_2_O_3_ was immersed in salt solutions with different concentrations for 24 h. When the complex action of cerium and copper was studied, the concentration of copper was controlled at 0.5 M and copper was mixed with different concentrations of cerium nitrate in solution.The presence of Cu on Al_2_O_3_ significantly improved the humic acid mineralization efficiency compared to that of pure Al_2_O_3_. A maximum TOC removal value of 49.72% was obtained at an oxidation time of 30 min when the impregnation concentration of Cu was 0.5 M; the removal rate was only 25.20% with pure Al_2_O_3_. When the immersion concentration of copper was 0.1 and 1 M, the TOC removal rates decreased to 34.77 and 31.38%, respectively. The effect of the cerium impregnation concentration was also studied when blending with copper. When 0.1 M cerium was mixed with a 0.5 M copper salt solution, the TOC removal rate increased to 54.79%, which was higher than those of the single metal loadings of Cu and Ce, namely, 49.72 and 49.07%, respectively. Similar results could be observed in that too high of an impregnation concentration of cerium would lead to a reduction in the TOC removal rate.

**Text S5 Effect of the hydroxyl radical scavenger**

To verify the role of ·OH in the catalytic ozonation of HA by Cu/Ce-Al_2_O_3_, tert-butyl alcohol (TBA) was added to the reaction system as a strong radical scavenger. Tert-butyl alcohol has a reaction rate constant of 6.0×10^8^ M^−1^ s^−1^ with hydroxyl radicals and one of only 3.0×10^−3^ M^−1^ s^−1^ with ozone. As shown in Fig. 3(b), the efficiency of HA degradation was significantly decreased to 40.38% at 30 min in the presence of 10 mM TBA compared to a 79.30% HA removal rate of the Cu/Ce-Al_2_O_3_/O_3_ process at 30 min. This finding indicated that the ·OH radical was indispensable to the reaction of HA with Cu/Ce-Al_2_O_3_/O_3_. Sole ozonation also involved the HO• reaction because the final HA degradation rate when TBA had been added was lower than that of ozone alone.

**Text S6 pH changes and metals leaching among the different process**

In the ozonation process, the pH values gradually decreased under different pH conditions. This result was due to the formation of a small amount of organic acids (such as formic acid and oxalic acid) in the ozonation process of HA, which mostly did not react with ozone and accumulated as final products. However, in the Cu/Ce-Al_2_O_3_/O_3_ process, the pH of the solution decreased during the first 10 min and then increased during the subsequent period of 10 to 30 min. The decrease in pH was related to the formation of small acids during the first 10 min. In catalytic ozonation, these small molecule acids, which weakly reacted with ozone, could react with the hydroxyl radicals produced in the catalytic process and be mineralized; therefore, the pH of the solution gradually increased during the period of 10-30 min. This phenomenon could be confirmed by the TOC removal rate in Cu/Ce-Al_2_O_3_/O_3_ processes (see Fig. 5(a)) and further showed that Cu/Ce-Al_2_O_3_/O_3_ could improve O_3_ decomposition to generate ·HO.

The effect of the initial pH of the solution on the leaching of metals in the Cu/Ce-Al_2_O_3_/O_3_ process was also studied. It can be seen from Table S4 that an increasing solution acidity or basicity would lead to copper leaching, and the dissolution rate of cerium was relatively low. The decrease in the number active sites on the surface of Cu/Ce-Al_2_O_3_/O_3_ reduced the quantity of surface hydroxyl groups, which depressed the generation of hydroxyl radicals. Therefore, Cu/Ce-Al_2_O_3_ presented the best catalytic activity under natural pH conditions (a pH of 7.0).

**Text S7 The analysis of FT-IR spectra in fingerprint area (1500-600 cm^-1^)**

HA had an absorption peak at 1378 cm^-1^, representing the C-H bending vibration of alkanes (1465-1340 cm^-1^), indicating that there were alkane structures present in HA. The absorption peaks of the SOP and COP samples at 1127 cm^-1^ were the C-O stretching vibrations of alcohol and phenol (1300-1000 cm^-1^) or the strong absorption peak of aliphatic compounds (1150-1060 cm^-1^). The absorption peaks of HA at 1030 cm^-1^ may have been the C-O stretching vibration of alcohols and phenols (1300-1000 cm^-1^) or the R-O stretching of aromatic ethers (1050-1000 cm^-1^). The absorption peaks of the SOP and COP samples at 997 cm^-1^ were the out-of-plane bending vibrations of olefin C-H (1000-675 cm^-1^). The SOP and COP samples had absorption peaks at 670 and 540 cm^-1^, respectively. The presence of carboxylic acid dimers, amines and amides may have led to absorption peaks near 700-600 cm^-1^. The peaks at 540 cm^-1^ indicated the C-X stretching of the aliphatic group of organic halides. HA had an anti-symmetric bending vibration peak of CH_3_ at 467 cm^-1^.

**Figure and Table**


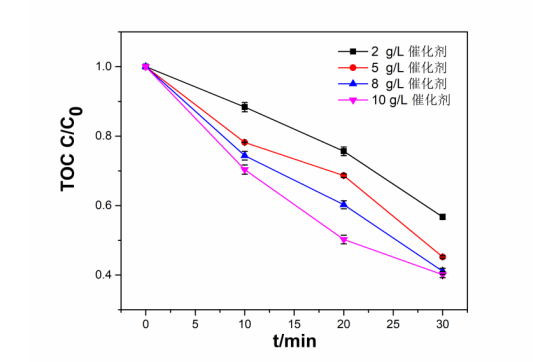


Fig. S1 Effect of catalyst dosage on catalytic ozonation of HA


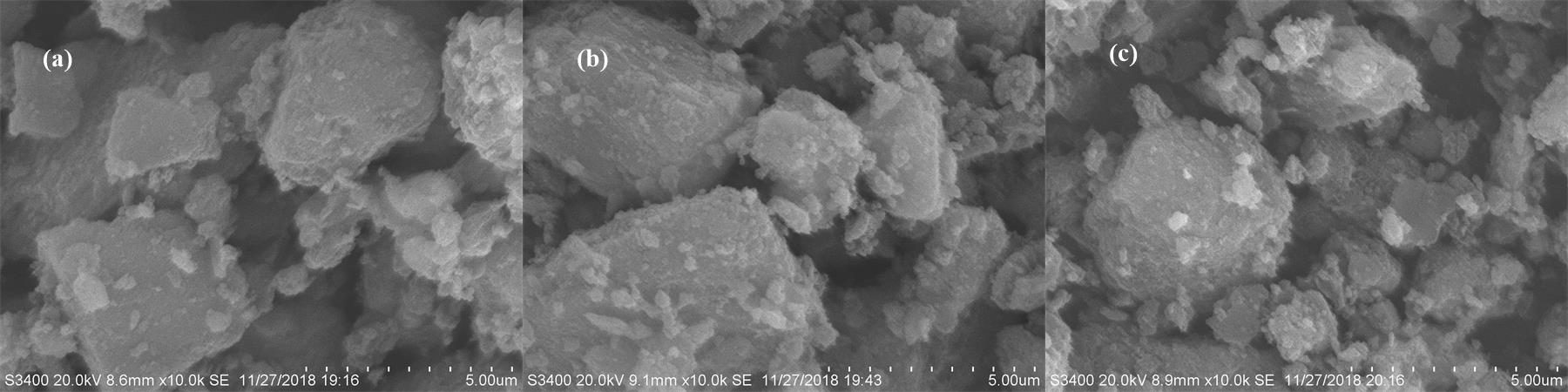


Fig. S2 SEM images of Al_2_O_3_ (a), Cu-Al_2_O_3_ (b) and Cu/Ce-Al_2_O_3_ (c)


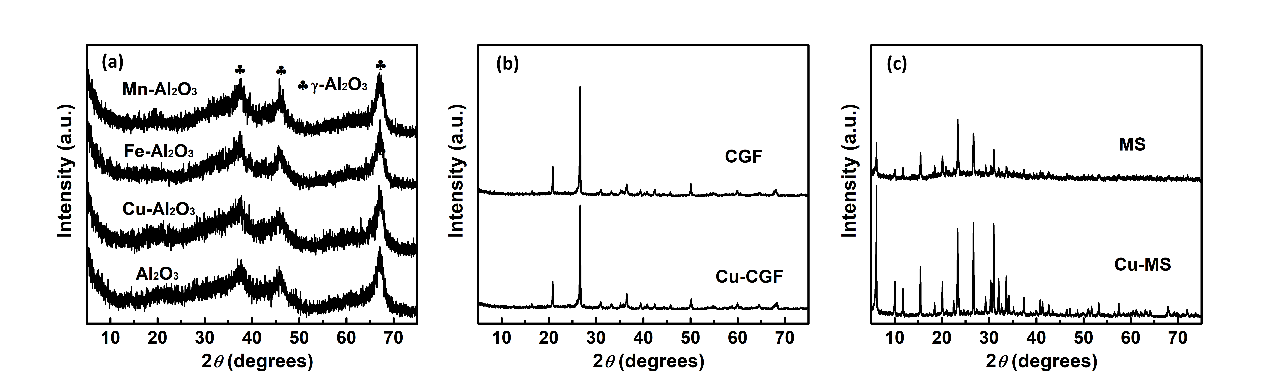


Fig. S3 XRD patterns: (a) Al_2_O_3_ catalysts, (b) GCF catalysts and (c) MS catalysts


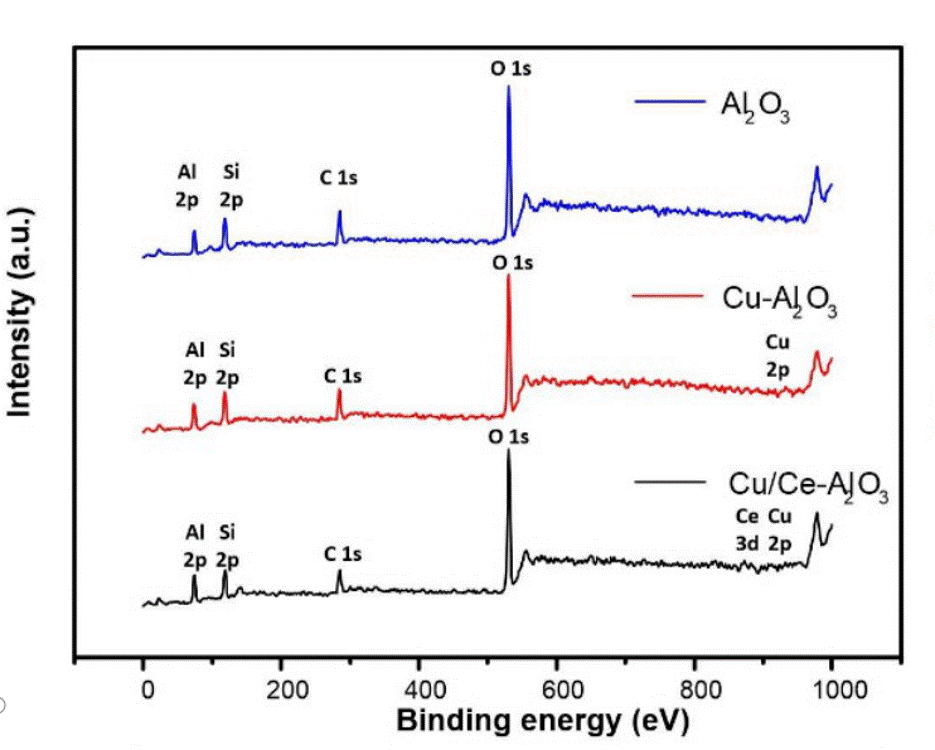


Fig. S4 Full-range XPS spectra of catalysts: Al_2_O_3_

**Table S1** Basic water quality of four kinds of biochemical tailwater

| **samples** | **WTTP1** | **WTTP2** | **WTTP3** | **WTTP4** |
| --- | --- | --- | --- | --- |
| pH | 8.01 | 8.13 | 8.76 | 8.48 |
| TOC (mg/L) | 42.31 | 45.5 | 87.82 | 4.3 |
| COD (mg/L) | 536 | 205 | 600 | 25 |
| BOD (mg/L) | 81.2 | 21.2 | 26.1 | 2.26 |
| colority | 108.48 | 142.05 | 447.71 | 6 |
| UV_254_ | 1.055 | 1.448 | 2.776 | 0.109 |
| HA (mg/L) | 57.58 | 97.72 | 215.66 | 7.21 |
| TN (mg/L) | 20 | 23.25 | 35.6 | 5.24 |

**Table S2** Physical properties of the catalyst support materials

| **Samples** | **BET surface area（m^2^/g）** | **Total pore volume（cm^3^/g）** | **Average pore diameter（nm）** | **pH_PZC_** |
| --- | --- | --- | --- | --- |
| CGF | 0.74 | 0.01 | 1.59 | 6.6 |
| MS | 484.63 | 0.30 | 2.52 | 5.5 |
| Al_2_O_3_ | 180.03 | 0.45 | 9.78 | 9.5 |

**Table S3 Atomic concentrations of the catalysts by** XPS

| **Samples** | **C 1s** | **O 1s** | **Al 2p** | **Si 2p** | **Cu 2p3** | **Ce 3d** | **Fe/Mn 2p** |
| --- | --- | --- | --- | --- | --- | --- | --- |
| Cu/Ce-Al_2_O_3_ | 25.42 | 47.64 | 24.87 | 1.23 | 0.69 | 0.15 |  |
| MS | 26.35 | 54.5 | 4.47 | 14.68 |  |  |  |
| CGF | 39.93 | 42.96 | 5.35 | 11.75 |  |  |  |
| Al_2_O_3_ | 28.43 | 49.28 | 20.71 | 1.58 |  |  |  |
| Cu-MS | 29.43 | 47.96 | 9.52 | 11.31 | 1.78 |  |  |
| Cu-CGF | 33.78 | 46.09 | 8.31 | 10.75 | 1.07 |  |  |
| Cu-Al_2_O_3_ | 27.92 | 47.29 | 22.44 | 1.68 | 0.67 |  |  |
| Fe-Al_2_O_3_ | 29.84 | 47.55 | 20.51 | 1.02 |  |  | 1.08 |
| Mn-Al_2_O_3_ | 23.63 | 50.81 | 23.85 | 1.16 |  |  | 0.55 |

**Table S4 Effect of the initial pH of the solution on the leaching of metals in the Cu/Ce-Al_2_O_3_/O_3_ process**.

| **Initial pH** | 5.0 | 7.0 | 9.0 |
| --- | --- | --- | --- |
| **c(Cu) (μg/L)** | 503 | 430 | 503 |
| **c(Ce) (μg/L)** | 37 | 58 | 66 |

**Table S5 The compounds identified analysis of humic acid (HA), catalytic ozonation process (COP) and sole ozonation process (SOP) samples**

| **Organic compounds** | **Chemical formula** | **HA** | | **COP** | | **SOP** | |
| --- | --- | --- | --- | --- | --- | --- | --- |
| **Full name** |  | **RPA**^a^ | **PA**^b^ | **RPA** | **PA** | **RPA** | **PA** |
| trans-13-Docosenamide | C_22_H_43_NO | 19.61 | 2.65E+06 | 14.56 | 8.00E+05 | 14.14 | 9.02E+05 |
| 13-Docosenamide, (Z)- | C_22_H_43_NO | 16.22 | 2.19E+06 | 10.24 | 5.62E+05 | 12.54 | 8.00E+05 |
| Erucic acid | C_22_H_42_O_2_ | 13.31 | 1.80E+06 | 11.66 | 6.41E+05 | 11.87 | 7.57E+05 |
| n-Hexadecanoic acid | C_16_H_32_O_2_ | 9.99 | 1.35E+06 | 15.31 | 8.41E+05 | 15.16 | 9.67E+05 |
| 9-Octadecenamide, (Z)- | C_18_H_35_NO | 7.91 | 1.07E+06 | 11.42 | 6.27E+05 | 12.64 | 8.06E+05 |
| Oleanitrile | C_18_H_33_N | 7.19 | 9.72E+05 | 8.23 | 4.52E+05 | 6.69 | 4.27E+05 |
| 1-Docosene | C_22_H_44_ | 4.73 | 6.40E+05 | ND | ND | 3.69 | 2.35E+05 |
| Phenol, 2,4-di-t-butyl-6-nitro- | C_14_H_21_NO_3_ | 3.86 | 5.23E+05 | 3.60 | 1.98E+05 | 3.86 | 2.46E+05 |
| Pentadecane, 2,6,10,14-tetramethyl- | C_19_H_40_ | 3.13 | 4.24E+05 | 3.82 | 2.10E+05 | ND^c^ | ND |
| Pentadecane, 2,6,10-trimethyl- | C_18_H_38_ | 2.95 | 4.00E+05 | 2.45 | 1.34E+05 | ND | ND |
| Cyclopentene, 1,2,3,4,5-pentamethyl- | C_10_H_18_ | 2.90 | 3.93E+05 | 1.34 | 7.34E+04 | ND | ND |
| Hexadecane, 2,6,10,14-tetramethyl- | C_20_H_42_ | 2.76 | 3.74E+05 | 2.47 | 1.36E+05 | 2.42 | 1.55E+05 |
| Pentadecane | C_15_H_32_ | 2.45 | 3.32E+05 | ND | ND | ND | ND |
| Cyclopentene, 1,2,3,3,4-pentamethyl- | C_10_H_18_ | 1.64 | 2.22E+05 | 3.63 | 1.99E+05 | 2.21 | 1.41E+05 |
| 1-Nonadecene | C_19_H_38_ | ND | ND | 5.03 | 2.76E+05 | ND | ND |
| Pentanoic acid, 2,2,4-trimethyl-3-carboxyisopropyl, isobutyl ester | C_16_H_30_O_4_ | ND | ND | 3.44 | 1.89E+05 | 3.19 | 2.03E+05 |
| Phenol, 2,4-bis(1,1-dimethylethyl)- | C_14_H_22_O | ND | ND | 1.86 | 1.02E+05 | 2.02 | 1.29E+05 |
| Hexadecane, 2,6,10-trimethyl- | C_19_H_40_ | ND | ND | ND | ND | 3.19 | 2.04E+05 |
| Octadecanoic acid | C_18_H_36_O_2_ | ND | ND | ND | ND | 3.00 | 1.91E+05 |
| Nonadecane | C_19_H_40_ | ND | ND | ND | ND | 2.54 | 1.62E+05 |

a, Peak area

b, Relative peak area (%)

c, ND, not detected

Table S6 Analysis of Humic Acid Substances in WTTP1 by GC-MS

| **Organic compounds** | **Chemical formula** | **HA** | | **COP** | | **SOP** | |
| --- | --- | --- | --- | --- | --- | --- | --- |
| **Full name** |  | **RPA**^a^ | **PA**^b^ | **RPA** | **PA** | **RPA** | **PA** |
| Phenol, 2,4-bis(1,1-dimethylethyl)- | C_14_H_22_O | 69.05 | 1.96E+07 | 7.82 | 9.70E+05 | 60.35 | 1.15E+07 |
| Pentadecane, 2,6,10,14-tetramethyl- | C_19_H_40_ | 1.28 | 3.62E+05 | 2.4 | 2.97E+05 | 1.91 | 3.63E+05 |
| Hexadecane, 2,6,10,14-tetramethyl- | C_20_H_42_ | 1.79 | 5.08E+05 | 3.74 | 4.63E+05 | 2.47 | 4.68E+05 |
| Pentadecane, 2,6,10-trimethyl- | C_18_H_38_ | ND | ND | 1.58 | 1.96E+05 | 1.28 | 2.43E+05 |
| 9-Octadecenamide, (Z)- | C_18_H_35_NO | ND | ND | ND | ND | 9.69 | 1.84E+06 |
| Total humic substabces |  | 72.11 | 2.05E+07 | 15.54 | 1.93E+06 | 75.69 | 1.44E+07 |

**Table S7 Analysis of Humic Acid Substances in WTTP2 by GC-M**S

| **Organic compounds** | **Chemical formula** | **HA** | | **COP** | | **SOP** | |
| --- | --- | --- | --- | --- | --- | --- | --- |
| **Full name** |  | **RPA**^a^ | **PA**^b^ | **RPA** | **PA** | **RPA** | **PA** |
| Phenol, 2,4-bis(1,1-dimethylethyl)- 2,4- | C_14_H_22_O | 77.31 | 2.47E+07 | 5.69 | 5.80E+05 | 57.27 | 1.30E+07 |
| Hexadecane, 2,6,10,14-tetramethyl- | C_20_H_42_ | 1.51 | 4.83E+05 | 5.15 | 5.25E+05 | 2.78 | 6.28E+05 |
| Pentadecane, 2,6,10,14-tetramethyl- | C_19_H_40_ | 0.98 | 3.13E+05 | 3.32 | 3.38E+05 | 1.75 | 3.95E+05 |
| Nonadecane | C_19_H_40_ | ND | ND | ND | ND | 0.66 | 1.50E+05 |
| Total humic substabces |  | 79.8 | 2.55E+07 | 14.16 | 1.44E+06 | 62.46 | 1.41E+07 |

**Table S8 Analysis of Humic Acid Substances in WT**TP3 by GC-MS

| **Organic compounds** | **Chemical formula** | **HA** | | **COP** | | **SOP** | |
| --- | --- | --- | --- | --- | --- | --- | --- |
| **Full name** |  | **RPA**^a^ | **PA**^b^ | **RPA** | **PA** | **RPA** | **PA** |
| 9-Octadecenamide, (Z)- | C_18_H_35_NO | 13.7 | 9.51E+05 | ND | ND | 47.44 | 3.25E+06 |
| n-Hexadecanoic acid | C_16_H_32_O_2_ | 6.8 | 4.72E+05 | ND | ND | ND | ND |
| Phenol, 2,4-bis(1,1-dimethylethyl)- | C_14_H_22_O | 5 | 3.47E+05 | ND | ND | ND | ND |
| Hexadecane, 2,6,10,14-tetramethyl- | C_20_H_42_ | 2.14 | 1.49E+05 | ND | ND | ND | ND |
| Octadecanoic acid | C_18_H_36_O_2_ | 1.97 | 1.37E+05 | ND | ND | ND | ND |
| Pentadecane, 2,6,10,14-tetramethyl- | C_19_H_40_ | 1.4 | 9.72E+04 | ND | ND | 2.22 | 1.52E+05 |
| Nonadecane | C_19_H_40_ | 0.94 | 6.52E+04 | 3.69 | 1.11E+05 | 1.74 | 1.19E+05 |
| Erucic acid | C_22_H_42_O_2_ | ND | ND | 6.96 | 2.10E+05 | 6.32 | 4.32E+05 |
| Oleanitrile | C_18_H_33_N | ND | ND | ND | ND | 6.76 | 4.63E+05 |
| Pentadecane, 2,6,10-trimethyl- | C_18_H_38_ | ND | ND | ND | ND | 1.27 | 8.72E+04 |
| Total humic substabces |  | 31.95 | 2.22E+06 | 10.66 | 3.21E+05 | 65.75 | 4.50E+06 |

Table S9 Analysis of Humic Acid Substances in WTTP4 by GC-MS

| **Organic compounds** | **Chemical formula** | **HA** | | **COP** | | **SOP** | |
| --- | --- | --- | --- | --- | --- | --- | --- |
| **Full name** |  | **RPA**^a^ | **PA**^b^ | **RPA** | **PA** | **RPA** | **PA** |
| Hexadecane, 2,6,10,14-tetramethyl- | C_20_H_42_ | 20.1 | 1.16E+05 | 14.18 | 7.46E+04 | 2.65 | 1.40E+04 |
| Erucic acid | C_22_H_42_O_2_ | 16.75 | 9.70E+04 | 13.8 | 7.26E+04 | 4.22 | 2.23E+04 |
| Pentadecane, 2,6,10-trimethyl- | C_18_H_38_ | 15.08 | 8.73E+04 | 8.28 | 4.36E+04 | 1.46 | 7.72E+03 |
| Cyclopentene, 1,2,3,4,5-pentamethyl- | C_10_H_18_ | 13.22 | 7.65E+04 | 6.07 | 3.20E+04 | ND | ND |
| 9-Octadecenamide, (Z)- | C_18_H_35_NO | ND | ND | ND | ND | 62.41 | 3.29E+05 |
| Oleanitrile | C_18_H_33_N | ND | ND | ND | ND | 7.59 | 4.00E+04 |
| Hexadecane, 2,6,10-trimethyl- | C_19_H_40_ | ND | ND | ND | ND | 2.97 | 1.57E+04 |
| Pentadecane, 2,6,10,14-tetramethyl- | C_19_H_40_ | ND | ND | 15.93 | 8.38E+04 | ND | ND |
| Total humic substabces |  | 65.14 | 3.77E+05 | 58.27 | 3.07E+05 | 81.3 | 4.29E+05 |

**Table S10 the removal efficiency of humic acid and other indicators in wastewater by catalytic ozonation**

|  | R1 | R2 | R3 | R4 |
| --- | --- | --- | --- | --- |
| HA | 52.62 | 57.2 | 54.8 | 38 |
| colority | 85.29 | 55.81 | 79.33 | 89.47 |
| UV_254_ | 64.17 | 38.15 | 54.76 | 20.35 |
| COD | 63.62 | 7.32 | 9.17 | 44 |
| TOC | 13.11 | 8.19 | 16.51 | 15.16 |
| GC-MS | 90.59 | 94.33 | 85.54 | 18.72 |
| SUVA | 58.72 | 32.63 | 45.76 | 6.08 |
| B/C | 132.56 | 536.17 | 45.95 | 35.11 |
| BOD | 15.39 | -489.62 | -32.57 | 24.34 |
